# Regulation of human Th9 cell differentiation by lipid modulators targeting PPAR-γ and acetyl-CoA-carboxylase 1

**DOI:** 10.3389/fimmu.2024.1509408

**Published:** 2024-12-23

**Authors:** Swetha Peesari, Jeremy P. McAleer

**Affiliations:** Department of Pharmaceutical Sciences, Marshall University School of Pharmacy, Huntington, WV, United States

**Keywords:** Th9 cells, IL-9, ACC1 acetyl-CoA carboxylase 1, PPAR-gamma, CD4 T cell

## Abstract

CD4 T cell activation induces dramatic changes to cellular metabolism for supporting their growth and differentiation into effector subsets. While the cytokines IL-4, TGF-β and IL-21 promote differentiation into Th9 cells, metabolic factors regulating this process remain poorly understood. To assess the role of lipid metabolism in human Th9 cell differentiation, naïve CD4 T cells were purified from blood of healthy volunteers and cultured in the presence or absence of compounds targeting PPAR-γ, acetyl-CoA-carboxylase 1 (ACC1), and AMP-activated protein kinase (AMPK) for four days. Th9 cell differentiation significantly increased PPARG expression, and the PPAR-γ agonist rosiglitazone suppressed IL-9 in a dose-dependent manner. The rosiglitazone-mediated suppression also occurred in the presence of the glucose metabolism inhibitor 2-deoxy-D-glucose, suggesting it was independent of glycolysis. On the other hand, the PPAR-γ antagonist GW9662 had no significant effect on IL-9 production. Next, the role of fatty acid synthesis was tested by treating cells with inhibitors of ACC1 (TOFA) or AMP-activated protein kinase (AMPK; dorsomorphin). We demonstrate reciprocal functions for these enzymes, as ACC1 inhibition substantially increased IL-9 production, whereas AMPK inhibition resulted in undetectable levels. TOFA also decreased expression of ACACA, the gene encoding ACC1, demonstrating regulation at the transcriptional level. Finally, combining TOFA treatment with exogenous oleic acid restored IL-9 back to the levels in control Th9 cultures, suggesting that ACC1 suppresses Th9 differentiation through fatty acid synthesis. Overall, our data demonstrate that lipid regulators associated with intracellular fatty acid accumulation suppress Th9 cell differentiation. These findings may have clinical implications for conditions associated with elevated IL-9 production.

## Introduction

CD4 T cells contribute to host defense and inflammation by producing cytokines in response to antigenic stimuli (1). Distinct subsets have been named Th1, Th2, Th9, Th17, Th22, Tfh and Treg based on their characteristic effector cytokines and transcription factors expressed. Th9 cells are one of the most recent subsets identified, although the production of IL-9 in mitogen-stimulated helper T cells was first described in 1988 (2). Subsequently, IL-4 and TGF-β were shown to be the induction cytokines for IL-9 (3), with IL-21 further amplifying its production (4). Individually, IL-4 and TGF-β are also required for Th2 and Treg differentiation, respectively, demonstrating their pleiotropic functions. Transcription factors associated with Th9 cells include PU.1, BATF, IRF4 and PPAR-γ, among others. These transcription factors are not unique to the Th9 subset (5), suggesting that other pathways are involved in Th9 differentiation. Delineating the mechanisms regulating IL-9 production may have implications for the treatment of allergies, inflammatory bowel diseases and tumors (6–8).

Naïve T cell activation is associated with metabolic changes to support their clonal expansion, resulting in a shift from oxidative phosphorylation to aerobic glycolysis (9). Metabolism also regulates T cell effector differentiation, and human Th9 cells were shown to have high glycolytic flux compared to other Th subsets (10). The role of lipid metabolism in Th9 cells is less well understood. While T cell activation increases fatty acid synthesis and/or uptake to support membrane synthesis (9), specific roles for lipid-regulating transcription factors and enzymes in this process have not been entirely elucidated. Of note, PPAR-γ promotes fatty acid uptake and lipolysis in CD4 T cells (11). This transcription factor is expressed in Th2, Th9 and Treg cells and enhances effector cell function (12–14). In addition, Acetyl-CoA-Carboxylase 1 (ACC1) promotes *de novo* fatty acid synthesis by converting acetyl-CoA into malonyl-CoA (15). ACC1 is regulated by AMP-activated protein kinase (AMPK) such that low ATP levels cause AMPK to phosphorylate ACC1, thereby suppressing fatty acid synthesis and increasing ATP production. A comprehensive understanding of how lipid metabolism regulates IL-9 may help us to better understand links between obesity, allergies and inflammation.

To determine the role of lipid metabolism in human Th9 cell differentiation, naïve CD4 T cells were cultured under Th9 conditions with several compounds targeting lipid metabolism. We identify reciprocal effects for AMPK and ACC1 on human Th9 differentiation, as inhibitors for these enzymes decreased and increased IL-9 production, respectively. The phenotype caused by ACC1 suppression was rescued by supplementation with oleic acid, demonstrating that *de novo* fatty acid synthesis suppresses IL-9. Further, we show the PPAR-γ agonist rosiglitazone suppresses IL-9 in a dose-dependent manner, while the antagonist GW9662 had no significant effect on IL-9 production. Collectively, our data points to a model in which accumulation of intracellular fatty acids in CD4 T cells through either *de novo* synthesis or cellular uptake suppresses IL-9. Thus, targeting metabolic pathways in immune cells may be beneficial in treating hypersensitivity reactions.

## Material and methods

### Naïve CD4 T cell purification and culture

Blood samples from healthy human volunteers were obtained from Zen-Bio, Inc (Research Triangle Park, NC). Each volunteer signed IRB or FDA informed consent forms validated by Pearl Pathways, LLC. Samples were processed according to Standard Operating Procedure managed Good Laboratory Practice protocols in compliance with all legal and ethical regulations. Peripheral blood mononuclear cells (PBMCs) were isolated from buffy coat by density gradient centrifugation. Briefly, concentrated blood (buffy coat) was diluted 1:3 in PBS, overlayed onto Ficoll-Paque PLUS (GE Healthcare) and centrifuged at 400 x g for 20 minutes with the brake off. The cloudy interface containing PBMCs was collected and washed with calcium- and magnesium-free HBSS, centrifuged (350 x g, 5 min) and resuspended in calcium- and magnesium-free PBS containing 2% FBS and 1 mM EDTA. Naïve CD4 T cells were purified with EasySep Human Naive CD4^+^ T cell isolation kit (StemCell Technologies, Vancouver, BC, Canada). Cell purity ranging from 77-97% was confirmed by flow cytometry. Following purification, cells were resuspended in RPMI 1640 supplemented with 25mM Hepes, 2mM L-Gln, 7.5% Fetal Bovine Serum (FBS), penicillin (50U/ml), and streptomycin (50μg/ml). Unless otherwise noted, additional D-glucose was added to a final concentration of 4.5 g/L. Cells were plated in 96-well plates at 1.1-2.0 x 10^5^ cells/well and stimulated with the human T Cell Activation/Expansion Kit from Miltenyi-Biotec (130-091-441) at a ratio of 2 cells per particle.

For T cell differentiation, the following recombinant human cytokines were added from R&D Systems: IL-4 (50 ng/mL), TGF-β (5 ng/mL), and IL-21 (25 ng/mL). Additional treatments included rosiglitazone (10μM, 40μM, Tocris Bioscience), GW 9662 (10μM, Tocris), 2-deoxy-D-glucose (2DG, 1mM, Tocris), AICAR (200-500μM, Tocris), dorsomorphin dihydrochloride (5-40μM), fatostatin A (10μM, Tocris), CBM 301940 (1μM, Tocris), 5-(Tetradecyloxy)-2-furoic acid (TOFA, 10μM, Sigma-Aldrich), oleic acid (5-20μM, Cayman Chemical), and peptide P60 (75-100μM, Abbiotec). Cells were incubated with these treatments for four days at 37°C and 5% CO2.

### Flow cytometry

On day four, cells were resuspended in RPMI 1640 media containing phorbol 12-myristate 13-acetate (PMA, 50 ng/mL, Fisher Scientific), ionomycin (750 ng/mL, Fisher Scientific) and brefeldin A (5 μg/mL, Tocris) for four hours. Cells were then fixed and permeabilized with eBioscience Foxp3/Transcription Factor Staining Buffer Set, and stained with CD4-Alexa 488 (clone OKT4, Biolegend), FoxP3-PE (clone 206D, Biolegend), and IL-9-APC (clone MH9A4, Biolegend). Cells were analyzed on an Acea Novocyte 2000R flow cytometer, with data analyzed using FlowJo software.

### ELISAs

On day four, supernatants were collected and analyzed for human IL-9 and IL-5 using commercially available kits from Biolegend (San Diego, CA).

### RNA purification and RT-PCR

On day 4, total RNA was purified from T cell cultures using TRIzol reagent (Ambion) and quantitated by spectrophotometry (Nanodrop Lite, Thermo Scientific). One microgram of RNA was used as a template for reverse transcription using iScript cDNA Synthesis Kit (Bio-Rad, Hercules, CA). Real-time PCR reactions were performed using TaqMan Gene Expression Master Mix and FAM-MGB-labelled primers for GAPDH (Hs02786624_g1), GATA3 (Hs00231122_m1), FOXP3 (Hs01085834_m1), PPARG (Hs01115513_m1), SPI1 (Hs02786711_m1), and ACACA (01046047_m1) (Applied Biosystems, Foster City, CA). PCR reactions were run on a QuantStudio 3 (Applied Biosystems) with the following conditions: 50°C for 2 min, 95°C for 10 min, and 40 cycles of 95°C for 15 sec and 60°C for 1 min. Gene expression was normalized to the housekeeping gene GAPDH and calculated using the delta-delta Ct method.

### Statistical analysis

Data is plotted as the mean +/- standard error of the mean (SEM). Statistical significance between control and experimental groups was determined using two-tailed Student’s t-tests, with the number of asterisks representing correspondingly lower p-values (*p < 0.05, **p < 0.01 and ***p < 0.001).

## Results

### IL-21 maximizes IL-9 production in the presence of IL-4 and TGF-β

**Figure 1:**
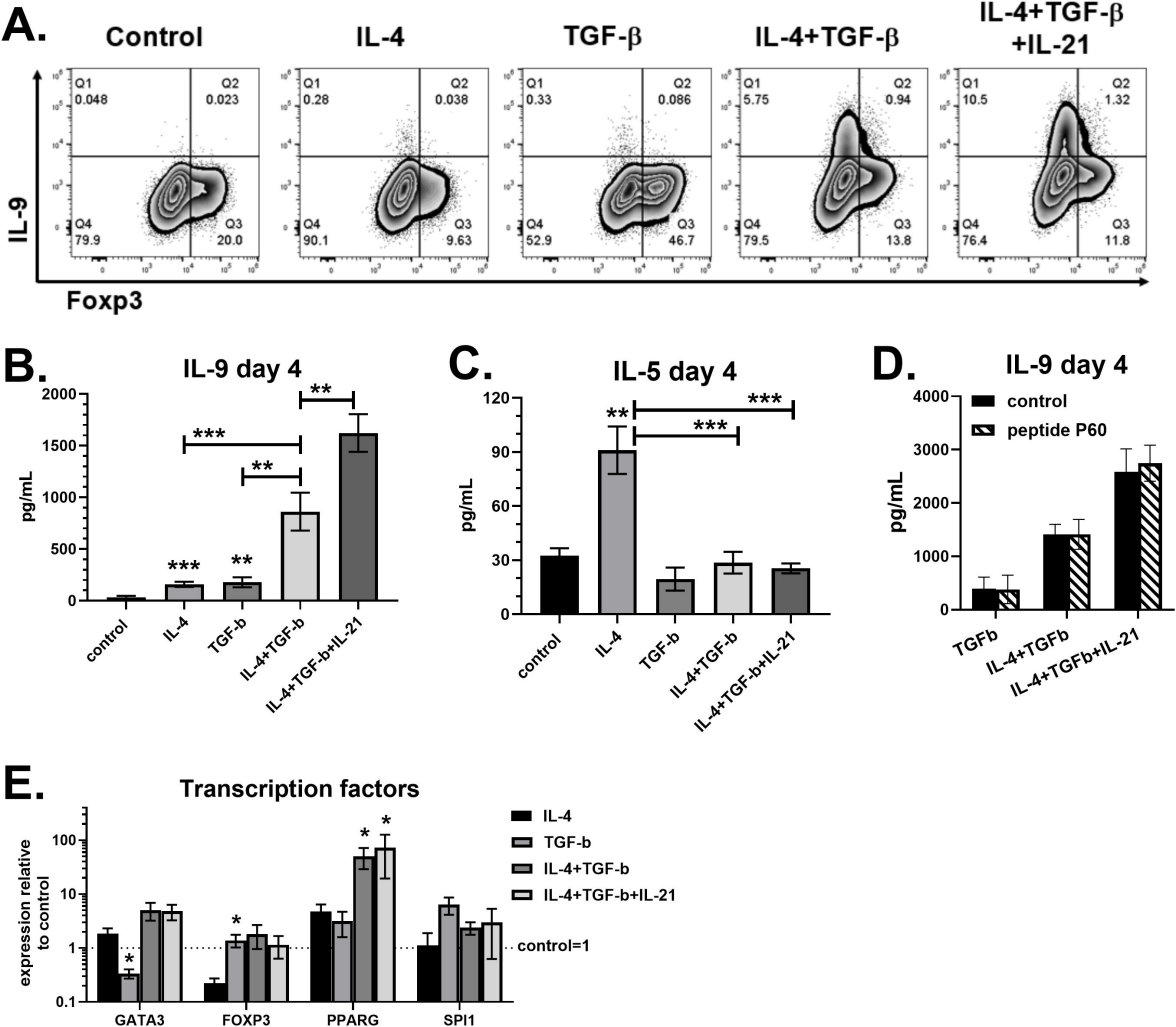
IL-21 maximizes IL-9 production in the presence of IL-4 and TGF-β. Naïve human CD4 T cells were cultured for 4 days with different combinations of IL-4 (50ng/mL), TGF-β (5ng/mL) and IL-21 (25ng/mL), as indicated. **(A)** On day 4, cells were restimulated with PMA plus ionomycin in the presence of brefeldin A and stained for IL-9 and Foxp3. Supernatants were measured for IL-9 **(B)** and IL-5 **(C)** by ELISA. Asterisks (*) represent significant differences compared to control cultures with no cytokines. **(D)** Cultures incubated for 4 days with or without peptide P60 (75-100μM) were assessed for IL-9 by ELISA. **(E)** Gene expression of GATA3, FOXP3, PPARG and SPI1, normalized to GAPDH, measured by PCR. Fold changes relative to control cultures with no cytokines are shown. Asterisks (*) represent significant differences compared to IL-4 treatment. Data in **(A-C)** are combined from four experiments with n=14-17. Data in **(D)** are combined from two experiments with n=2-3. Data in **(E)** are combined from three experiments with n=3-4.

Th9 cell differentiation is dependent on cytokines that also promote Th2 (IL-4) or Treg (TGF-β) differentiation (3). To assess their effects on human cells, naïve CD4 T cells were purified from peripheral blood of healthy volunteers and cultured for four days with activator beads targeting CD3, CD28 and CD2. Control cultures incubated without cytokines contained approximately 20 percent Foxp3^+^ cells on day 4 (**Figure 1A**), most likely due to anti-CD3 and anti-CD28 stimulation (16). Treatment with IL-4 alone significantly increased IL-5 production, consistent with Th2 cell differentiation, while TGF-β increased Foxp3 expression as expected (**Figures 1A, C**). While these cytokines alone produced low but detectable IL-9, the combination of IL-4 and TGF-β substantially increased the percent of CD4 T cells producing IL-9 (>3%) and concentrations in supernatants (>800pg/mL) (**Figure 1**). This was associated with IL-4 suppressing Foxp3 in cultures containing TGF-β, and TGF-β suppressing IL-5 in cultures containing IL-4 (**Figures 1A, C**). As expected (4), adding IL-21 to cultures containing both IL-4 and TGF-β nearly doubled the amount of IL-9 detected by flow cytometry and ELISA. Further, PPARG expression positively correlated with IL-9 production, as previously reported (12). Although PPARG was detected in each of the treatment conditions, the highest PPARG levels were measured in cultures containing both IL-4 and TGF-β (**Figure 1E**). The combination resulted in 10-15-fold increases in PPARG expression compared to single cytokine treatment. On the other hand, the gene encoding PU.1, SPI1, was not significantly affected by any treatment. Since a distinct Foxp3^+^ population was detected in Th9 cell cultures (**Figure 1A**), we next tested their role with the Foxp3 inhibitor peptide P60. Our data show that incubation with peptide P60 had no effect on IL-9 in cultures containing TGF-β (**Figure 1D**), demonstrating that Foxp3^+^ cells were not suppressing Th9 differentiation. Overall, the robust IL-9 production in response to IL-4, TGF-β and IL-21 is associated with elevated PPARG expression.

### PPAR-γ stimulation suppresses IL-9 in human CD4 T cells

**Figure 2:**
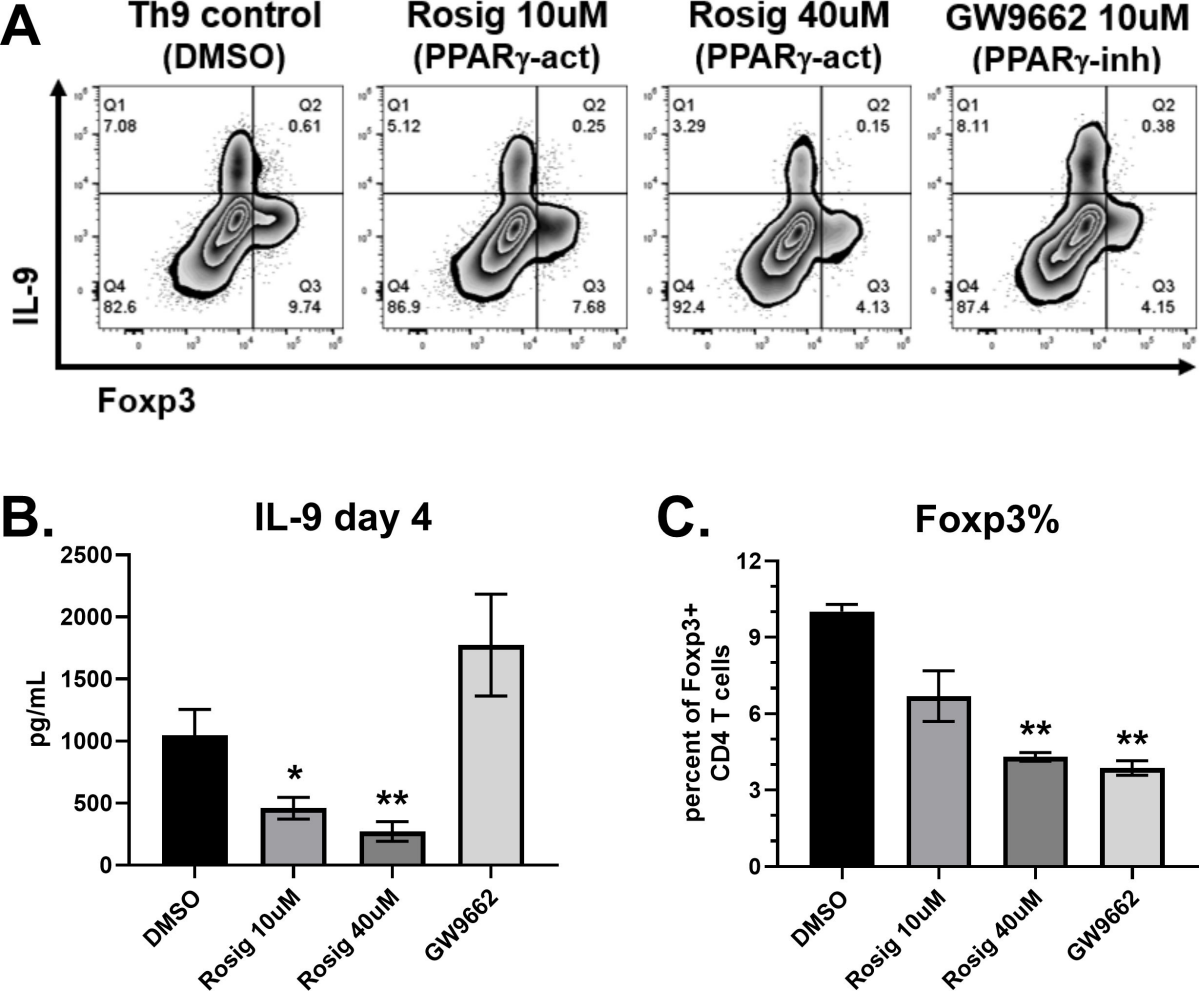
PPAR-γ stimulation suppresses IL-9 production. **(A)** Flow cytometry plots of IL-9 versus Foxp3 in response to treatments on day 4, as indicated. **(B)** IL-9 production measured by ELISA on day 4 supernatants in response to each treatment, as indicated. **(C)** Percent of CD4 T cells staining positive for Foxp3, measured by flow cytometry. Data in **(B)** are combined from 3 experiments with n=11. Data in **(C)** are combined from two experiments with n=2. act, activator; inh, inhibitor; Rosig, Rosiglitazone. Asterisks represent statistically significant differences compared to the DMSO group, with *p < 0.05 and **p < 0.01.

T cell activation results in metabolic changes to support the energy production and biomass accumulation required for their clonal expansion and effector cell differentiation. Lipid metabolism is regulated in part by cellular energy balance, enzymes (AMPK, ACC1) and transcription factors (SREBP, PPAR-γ). We first assessed the role of PPAR-γ since human Th9 cells expressed the highest levels of this transcription factor (**Figure 1E**). Naïve human CD4 T cells were cultured under Th9 conditions (IL-4, TGF-β, IL-21) for 4 days. In order to maximize IL-9 production, IL-21 (25ng/mL) and additional glucose were added to media (4.5 g/L final concentration), as previously described (4, 10). As expected, the Th9 group treated with DMSO control produced robust amounts of IL-9, resulting in >7 percent of CD4 T cells staining positive and average concentrations of 1.0 ng/mL in supernatants (**Figure 2**). A separate population of IL-9^-^ Foxp3^+^ cells was also observed in each condition. The PPAR-γ agonist rosiglitazone suppressed IL-9 in a dose-dependent manner, with 40μM reducing concentrations by 74 percent (**Figure 2B**). The PPAR-γ antagonist GW9662 did not significantly affect IL-9, although a trend towards increased production was observed. Both rosiglitazone and GW9662 decreased Foxp3^+^ percentages by approximately 60 percent, demonstrating a suppressive effect of PPAR-γ modulators on Treg differentiation under these conditions. Therefore, the ratio of Th9:Treg cells was largely unaffected by rosiglitazone, but increased by GW9662. Overall, PPAR-γ stimulation suppressed human Th9 cell differentiation whereas PPAR-γ inhibition had no significant effect.

### Rosiglitazone suppresses IL-9 independently of glycolysis

**Figure 3:**
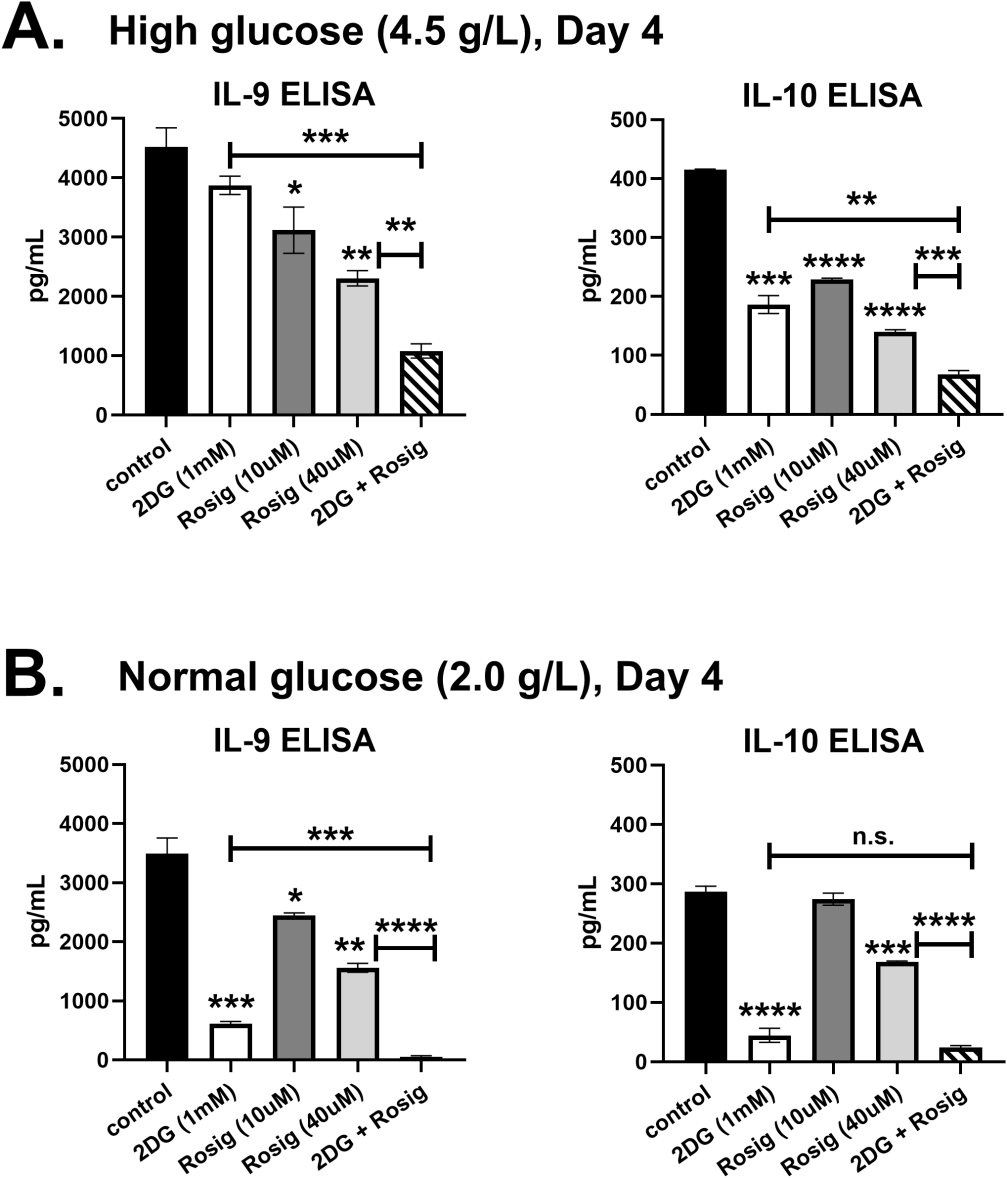
Rosiglitazone suppresses IL-9 independently of glycolysis. Naïve CD4 T cells were cultured in Th9 media (IL-4, TGF-β, IL-21) under **(A)** high glucose (4.5 g/L) or **(B)** normal glucose (2.0 g/L) conditions for 4 days. Cells were treated with 2DG (1mM), 10μM rosiglitazone, 40μM rosiglitazone, or 2DG plus 40μM rosiglitazone, as indicated. Supernatants were measured for IL-9 and IL-10 by ELISA. Data are from one experiment, representative of two independent experiments, with n=3. Asterisks represent statistically significant differences compared to the Control group, or between the groups shown by brackets, with *p < 0.05, **p < 0.01, ***p < 0.001 and ****p < 0.0001.

PPAR-γ is an insulin sensitizer and has been shown to upregulate GLUT4 on cell surfaces, enhancing glucose transport (17). Since activation-induced glycolysis was shown to be required for IL-9 production (10), the suppressive effect of rosiglitazone on IL-9 may be independent of the glycolytic pathway. To test this, T cells were cultured with rosiglitazone in the presence or absence of the glycolysis inhibitor 2-deoxy-D-glucose (2DG; 1mM). As expected, rosiglitazone suppressed IL-9 in a dose-dependent manner under high glucose (4.5 g/L) and normal glucose (2.0 g/L) conditions (**Figure 3**). Notably, incubating cells with 40μM of rosiglitazone resulted in a substantial 50 percent reduction in IL-9. Treatment with 2DG had no significant effect on IL-9 under high glucose conditions (**Figure 3A**), but decreased IL-9 by 80 percent in normal glucose cultures (**Figure 3B**). Importantly, the combination of 2DG plus rosiglitazone was synergistic compared to either treatment alone, resulting in significantly lower IL-9 levels in both high glucose and normal glucose conditions. The anti-inflammatory cytokine IL-10 was also suppressed by rosiglitazone and 2DG (**Figure 3**). Although these data do not exclude the possibility that rosiglitazone could be acting independently of PPAR-γ (e.g. decreasing cell viability in the presence of 2DG), they suggest that a thiazolidinedione medication can suppress IL-9 independently of glycolytic activity.

### ACC1 suppresses IL-9 production through *de novo* fatty acid synthesis

**Figure 4:**
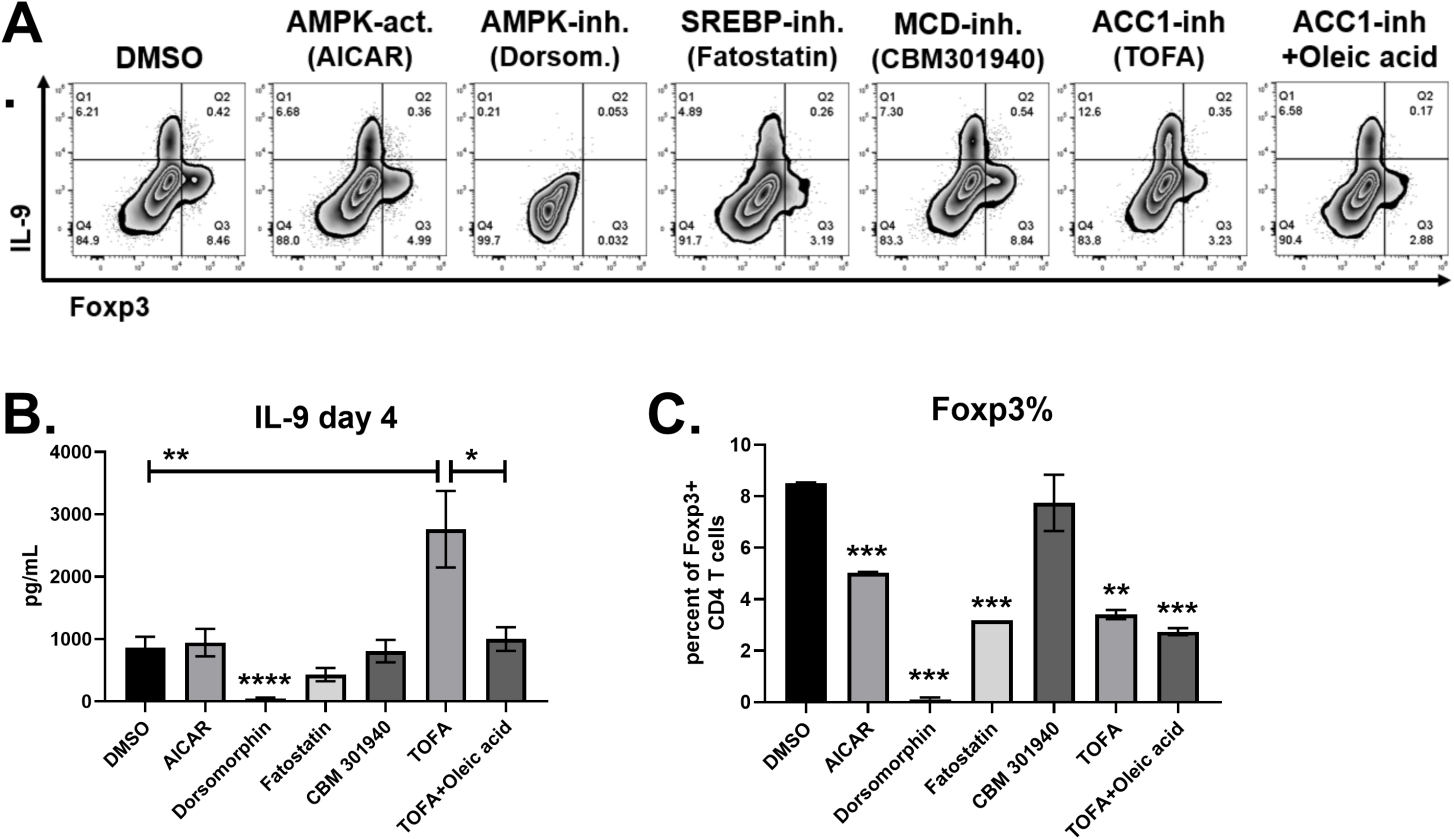
IL-9 is suppressed by ACC1 and exogenous oleic acid. **(A)** Flow cytometry plots of IL-9 versus Foxp3 in response to treatments on day 4, as indicated. **(B)** IL-9 production measured by ELISA on day 4 supernatants in response to each treatment, as indicated. **(C)** Percent of CD4 T cells staining positive for Foxp3, measured by flow cytometry. Data in **(B)** are combined from 3 experiments with n = 7-11. Data in **(C)** are combined from two experiments with n=2 or 1 (fatostatin only). act, activator; inh, inhibitor; Dorsom, Dorsomorphin dihydrochloride. Asterisks represent statistically significant differences compared to the DMSO group, or between the groups shown by brackets, with *p < 0.05, **p < 0.01, ***p < 0.001 and ****p < 0.0001.

Next, naïve CD4 T cells were cultured with Th9 inducing cytokines in the presence of compounds targeting AMPK, ACC1 and SREBP. The AMPK agonist AICAR had no effect on IL-9 production, but significantly decreased Foxp3^+^ cells (**Figure 4**). The AMPK inhibitor dorsomorphin dihydrochloride completely suppressed both IL-9 and Foxp3, demonstrating an indispensable role for AMPK in CD4 T cell differentiation. The ACC1 inhibitor TOFA doubled the percentage of T cells producing IL-9 (**Figure 4A**), and tripled IL-9 levels in supernatants (**Figure 4B**), demonstrating that ACC1 suppresses human Th9 cell differentiation. In addition, TOFA decreased Foxp3^+^ cells by 45 percent (**Figure 4C**). Since ACC1 promotes fatty acid synthesis, we tested if adding fatty acids to media can reverse the effect of TOFA treatment. Indeed, oleic acid restored IL-9 in TOFA-treated cells to similar levels found in the DMSO control group (**Figure 4**). This effect was specific to IL-9, as Foxp3 expression was unaffected by oleic acid. The enzyme malonyl-CoA decarboxylase (MCD) counteracts ACC1 by converting malonyl-CoA back into acetyl-CoA. Treatment with the MCD inhibitor CBM 301940 had no significant effect on IL-9 or Foxp3 expression, suggesting that MCD was not involved in Th9 differentiation. Fatty acid and cholesterol synthesis are positively regulated by the SREBP transcription factor. The SREBP inhibitor fatostatin significantly decreased Foxp3^+^ cells without affecting IL-9, although there was a trend towards decreased IL-9 (**Figure 4**). Overall, these data suggest that ACC1-induced fatty acid synthesis suppresses human Th9 cell differentiation.

### Effect of lipid modulators on ACC1 and PPAR-γ gene expression

**Figure 5:**
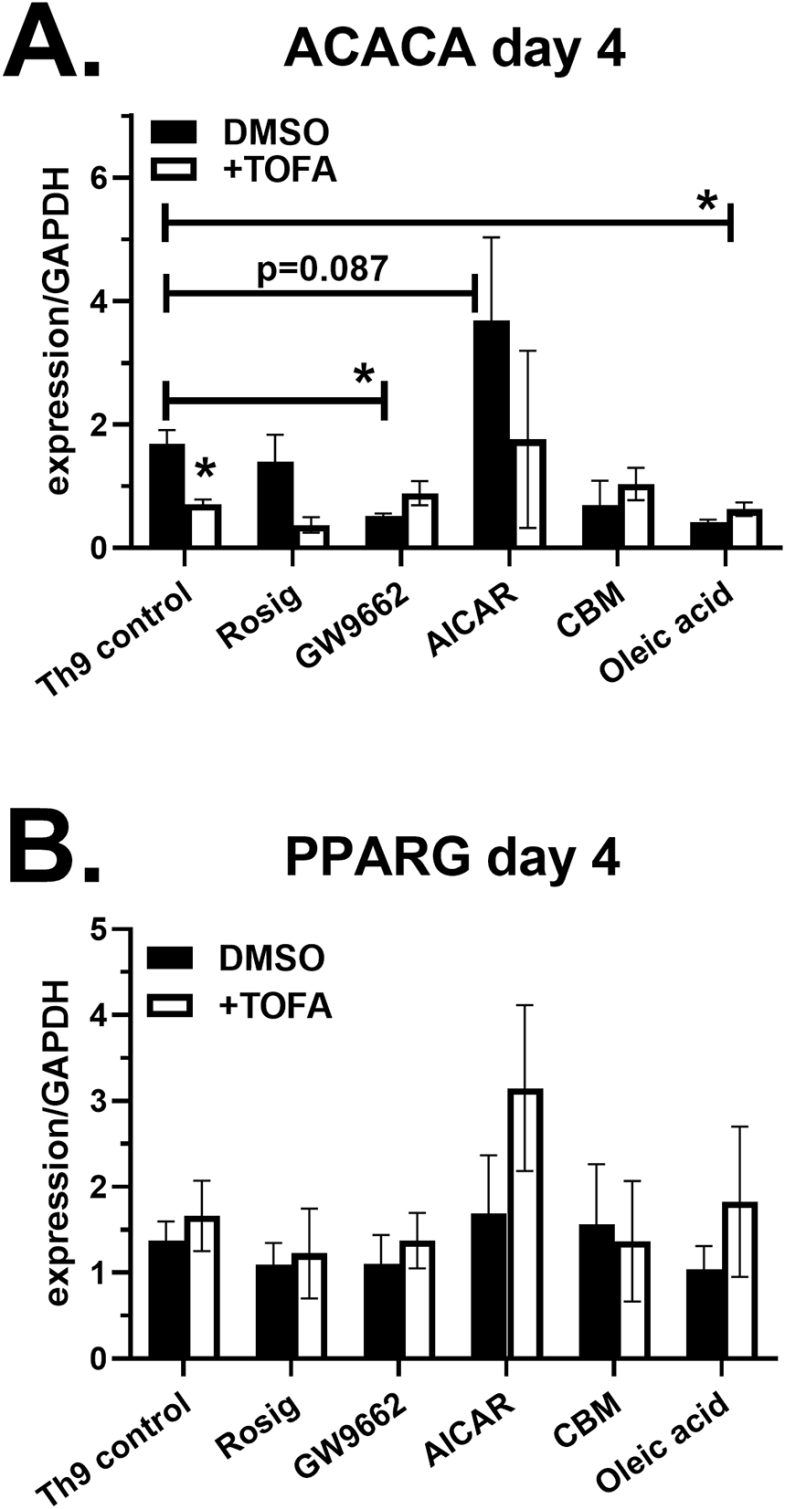
Lipid modulators regulate ACC1 gene expression. Naïve CD4 T cells were cultured under Th9 conditions in the presence of lipid modulators, as indicated. On day 4, RNA was collected, converted to cDNA and analyzed for gene expression of ACACA **(A)** and PPARG **(B)** by real-time PCR. Data are combined from 2 experiments with n=2-4. Asterisks represent statistically significant differences between the Th9 control treated with DMSO and indicated group, with *p < 0.05.

To determine if lipid modulators regulate gene expression for ACC1 (ACACA) and PPAR-γ (PPARG), RNA from Th9 cell cultures was collected on day 4, converted to cDNA and analyzed by real-time PCR. ACACA expression was significantly decreased by GW9662 and TOFA, demonstrating that inhibitors for PPAR-γ and ACC1 both have suppressive effects on ACC1 gene expression (**Figure 5A**). In addition, oleic acid decreased ACACA, demonstrating that fatty acid uptake also has a suppressive effect. The AMPK agonist AICAR resulted in a trend increased ACACA levels; however, the modest increases were not statistically significant. In contrast to ACACA, we did not observe any significant changes to PPARG expression in response to the treatments (**Figure 5B**), demonstrating that lipid modulators were not regulating PPAR-γ at the transcription level.

## Discussion

T cell activation results in metabolic changes to support the energy production and biomass accumulation required for clonal expansion and effector cell differentiation. This has been well characterized for glucose metabolism, with Akt and mTOR playing critical roles in driving aerobic glycolysis (9). *In vivo*, several studies have demonstrated a requirement for mTOR in Th9-mediated inflammation (18–20). Accordingly, Th9 cells were found to be highly glycolytic compared to Th1 and Th2 subsets due to enhanced mTORC1 activation (10). This was attributed to PPAR-γ increasing expression of the lactate transporter MCT-1 in T cells treated with IL-4 and TGF-β. While that study found pre-incubating cells with GW9662 for 48h prior to *in vitro* activation suppressed IL-9, we did not observe suppression when GW9662 was given at the same time as Th9-inducing cytokines. Rather, the PPAR-γ agonist rosiglitazone suppressed IL-9 in a dose-dependent manner (**Figure 2**). The suppressive effect of rosiglitazone was also observed in the presence of 2DG (**Figure 3**), suggesting that PPAR-γ agonists may have glycolysis-independent effects on Th9 differentiation. The elevated expression of PPARG in Th9 cells compared to other subsets suggests they are particularly sensitive to metabolic regulation by PPAR-γ.

The function of PPAR-γ has been characterized in adipocytes and leukocytes. As a member of the nuclear receptor superfamily of peroxisomal proliferators activated by fatty acids, PPAR-γ regulates genes for adipogenesis, lipid metabolism, glucose homeostasis and inflammation (21–23). Accordingly, PPAR-γ is a therapeutic target in type 2 diabetes mellitus and inflammatory bowel diseases. Several reports have detailed the functions of PPAR-γ in CD4 T cells. In response to antigen and costimulation, PPAR-γ promotes fatty acid uptake and lipolysis, enhancing proliferation (11). STAT6 upregulates PPARG in response to IL-4, enhancing IL-5 production along with lipid metabolism (13). *In vivo*, PPARG expression in CD4 T cells contributes to Th2-mediated inflammation in the intestine, lungs and skin (24–26). Human Th9 cells have been characterized as a subpopulation of PPAR-γ^+^ Th2 cells that produce IL-9, IL-5 and IL-13 (12). *In vitro* primed Th9 cells had reduced IL-9 secretion when they were pre-incubated with the PPAR-γ inhibitor GW9662 prior to undergoing Th9 differentiation (12). In contrast, we did not observe a decrease in IL-9 when cells were treated with GW9662 at the same time as Th9-inducing stimuli. We also used IL-21 in cultures whereas Micosse, et al. did not. Rather, we observed a dose-dependent decrease in IL-9 using the PPAR-γ agonist rosiglitazone (**Figure 2**). Future studies are necessary to determine if rosiglitazone suppresses IL-9 through the uptake of extracellular fatty acids, and to confirm that the effects of rosiglitazone are mediated by PPAR-γ. Our data are consistent with reports showing therapeutic effects of PPAR-γ agonists in atopic dermatitis and psoriasis (24, 27–29). Regulatory T cells (Tregs) also express PPAR-γ; however, thiazolidinediones increased Foxp3 expression and Treg differentiation independently of PPAR-γ (14). We found that rosiglitazone decreases Foxp3 under Th9 priming conditions, suggesting the presence of IL-4 prevents rosiglitazone from upregulating Foxp3. *In vivo*, PPAR-γ expression in Tregs protects against colitis, psoriasis, graft-versus-host disease and insulin resistance (14, 30–33), demonstrating anti-inflammatory properties. Overall, PPAR-γ expression in several cell types contributes to its complex functions in metabolism and inflammation during various disease states.

T cell activation increases the demand for fatty acids, requiring the coordinated action of several enzymes and transcription factors (9). Acetyl-CoA Carboxylase 1 (ACC1) facilitates *de novo* fatty acid synthesis by catalyzing the ATP-dependent carboxylation of acetyl-CoA into malonyl-CoA, which is then utilized by fatty acid synthase to produce mid- and long-chain fatty acids (15). The activity of ACC1 is suppressed by AMPK through phosphorylation when the cellular AMP/ATP ratio is elevated. Therefore, AMPK increases cellular ATP production by promoting fatty acid oxidation. Several studies have assessed the function of ACC1 in T cells. Diet-induced obesity increases ACC1 expression in memory CD4 T cells, driving IL-17 production *in vitro* and Th17-dependent pathology *in vivo* (34). Pharmacologic suppression of ACC1 not only decreased IL-17, but increased Foxp3 expression and Treg differentiation (35). Accordingly, mice with T cell-specific ACC1 deficiency are protected from experimental asthma, psoriasis and colitis, but are more susceptible to infections (36–39). These pro-inflammatory effects of ACC1 appear to be mediated by glycolytic and oxidative metabolic reprogramming (40). Here, we identified an anti-inflammatory function of ACC1 in suppressing IL-9. Specifically, TOFA treatment not only increased IL-9, but decreased Foxp3 percentages in cultures, demonstrating a pro-inflammatory shift in T cell cultures. Although TOFA treatment increased IL-9 on a per cell basis, we did not measure cell growth or proliferation. The enhancement of IL-9 production by TOFA was restored with exogenous oleic acid, suggesting that fatty acid synthesis and uptake both contribute to IL-9 suppression. TOFA is a competitive inhibitor of ACC1, and here we show that it also decreases ACACA expression, the gene encoding for ACC1 (**Figure 5**). Exogenous oleic acid and GW9662 treatment also suppressed ACACA, demonstrating its metabolic regulation. Since protein expression was not examined, it will be important to discern how Th9-inducing cytokines and lipid modulators affect total and phosphorylated ACC1 forms in primary CD4 T cells. Previous studies demonstrated that a PPAR-γ agonist suppresses ACC1 in Th17 cells (41), and ACC1-deficiency in iNKT cells reduces PPAR-γ expression (37), suggesting there is cross-regulation between these metabolic pathways. In contrast to TOFA, the AMPK agonist AICAR did not increase IL-9, possibly due to its enhancement of fatty acid uptake (42). Since murine studies have previously found that ACC1 suppression can either enhance or reduce memory CD4 T cell generation depending on the conditions (43, 44), future studies are necessary to determine the impact of ACC1 suppression on Th9 responses *in vivo*.

Mechanistically, elevated acetyl-CoA levels in T cells following ACC1 suppression was shown to promote calcium influx and NFAT1 nuclear translocation (45), which may play a role in the increased IL-9 production we observed. ACC1 suppression also increases histone acetylation (46), which can have widespread effects on gene expression. Further, ACC1 protects cancer cells from oxidative stress, enhancing their survival (47). Although the role of oxidative stress in Th9 differentiation is not clear, IL-9R expression in cutaneous T cell lymphoma cells reduces oxidative stress in the presence of IL-9 (48). Thus, it is intriguing to hypothesize that the IL-9 resulting from TOFA treatment improves Th9 cell survival by alleviating oxidative stress, as Th9 cells are known to express high levels of IL-9R (49). Elucidating the mechanisms contributing to IL-9 production in the context of ACC1 suppression will yield insights into the precise roles of lipid metabolism in regulating inflammation.

Th9 and Treg subsets are both dependent on TGF-β for their development, and Foxp3^+^ IL-9^-^ cells were observed in our Th9 cultures. Incubation with a Foxp3 inhibitor had no effect on IL-9 production (**Figure 1D**), suggesting that Tregs were not suppressing IL-9 under these conditions. Intriguingly, several compounds targeting PPAR-γ, ACC1, AMPK and SREBP decreased Foxp3 percentages, and further studies are necessary to examine the underlying mechanisms. Thiazolidinedione treatment *in vitro* or *in vivo* has been shown to increase the percent of CD4 T cells expressing Foxp3 (14, 30, 50), possibly by enhancing TGF-β receptor or IL-2 receptor expression (51). Regulation of Foxp3 expression by PPAR-γ agonists has not been well studied in Th9 culture conditions. The suppression of Foxp3 by rosiglitazone in Th9 cultures may involve GATA3, as the GATA3-inducing cytokine IL-4 suppresses Foxp3 and PPAR-γ enhances Th2 cell effector function (13, 49, 52). ACC1-mediated fatty acid synthesis promotes Th17 differentiation, as *in vitro* treatment with ACC1 inhibitors suppress IL-17 and increase Foxp3 (35). Another study found that a PPAR-γ agonist regulates ACC1 by promoting AMPK activation in Th17 cells, increasing Foxp3 expression (41). These Th17 studies contrast with our findings using Th9 conditions, whereby TOFA treatment decreased Foxp3 percentages (**Figure 4**). Since the primary inducers of Foxp3 in human CD4 T cells are anti-CD3/CD28 stimulation and exogenous TGF-β (16, 53), lipid modulators may regulate Foxp3 through altered function of T cell surface receptors or transcription factors required for Th9 cell function.

**Figure 6:**
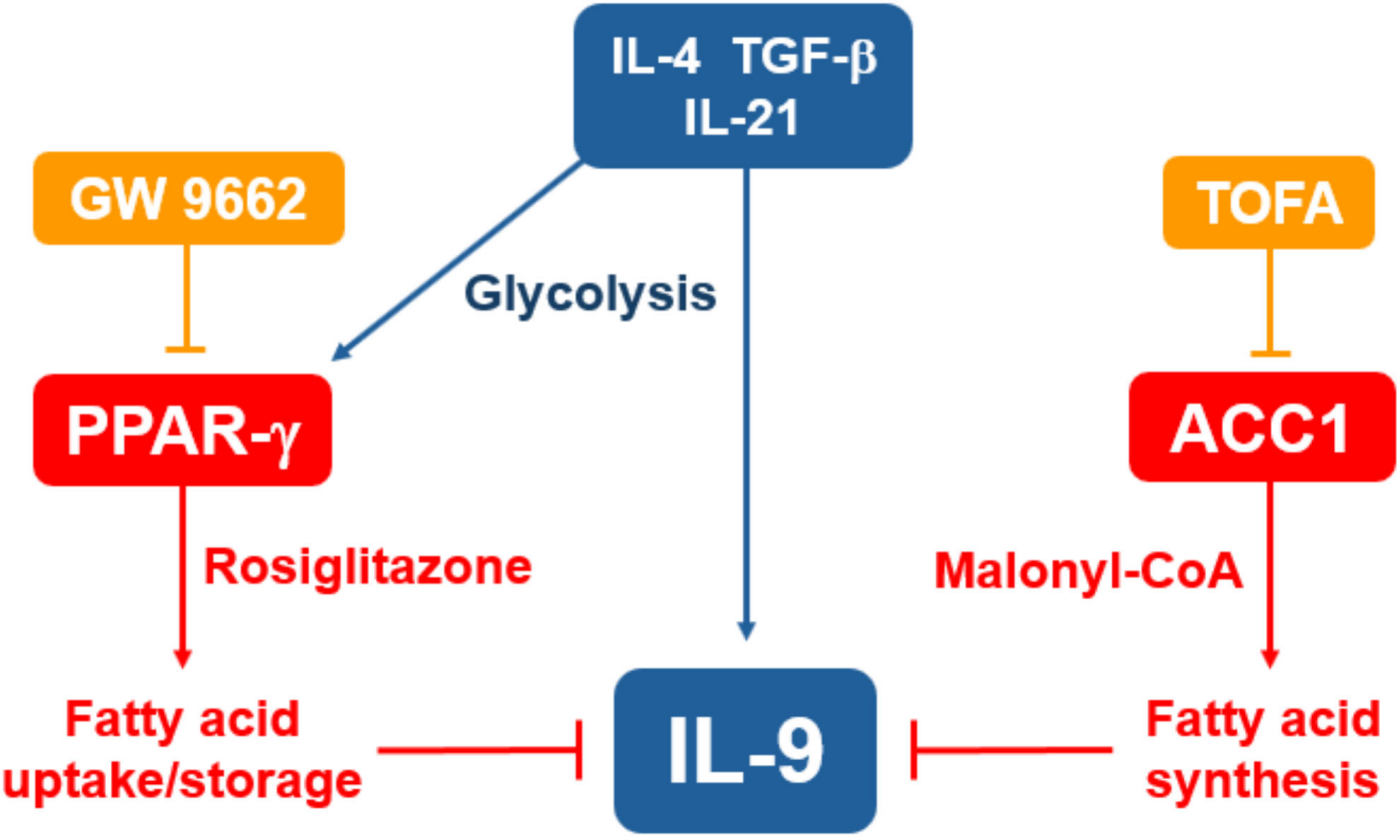
Suppression of IL-9 through lipid metabolism. Treating naïve CD4 T cells with IL-4, TGF-β and IL-21 induces robust IL-9 production and expression of PPAR-γ. Treatment with the PPAR-γ agonist rosiglitazone is expected to increase fatty acid uptake and storage, leading to suppression of IL-9. Treatment with the ACC1-inhibitor TOFA suppresses fatty acid synthesis, leading to increased IL-9 production.

The clinical relevance of our findings remain an important area for investigation. Pathogenic roles for IL-9 have been identified in several allergic and autoimmune diseases, including atopic dermatitis, asthma, colitis and multiple sclerosis (54). Although PPAR-γ is expressed in both pro- and anti-inflammatory T cell subsets, several diseases seem to benefit from treatment with PPAR-γ agonists (28, 55, 56). This may be due to the downregulation of Th2 and Th9 effector cytokines, or from enhanced suppressive function of Tregs. As a central regulator of *de novo* fatty acid synthesis, ACC1 expression in T cells contributes to some of the diseases that have been associated with IL-9, such as asthma, psoriasis and colitis (36–38). Therefore, pharmacologic suppression of ACC1 *in vivo* may be able to provide therapeutic benefits without necessarily inducing IL-9 mediated pathology. The roles of PPAR-γ and ACC1 in T cell metabolism suggest that medications targeting these molecules may be utilized in obesity-related inflammation. For instance, treating type 2 diabetes patients with thiazolidinediones could have the dual effects of improving insulin sensitivity and helping to alleviate the chronic inflammation associated with this patient population. In support, a mouse model found that diet-induced obesity increased the severity of atopic dermatitis in an IL-17 dependent manner, and that rosiglitazone suppressed IL-17 and restored the effectiveness of anti-IL-4 and anti-IL-13 therapy (24). Obesity in humans is associated with elevated ACACA expression and IL-17 production from memory CD45RO^+^ CD4 T cells (34). Although ACC1 suppression may be effective in downregulating Th17 responses, this treatment has been associated with hypertriglyceridemia, possibly restricting its therapeutic value (57). In summary, we demonstrated that human Th9 cell differentiation is suppressed by lipid modulators targeting ACC1 and PPAR-γ, implicating *de novo* fatty acid synthesis and possibly fatty acid uptake in suppressing IL-9 production *in vitro* (**Figure 6**). Our data suggest that medications targeting lipid metabolism may have significant effects on allergies and autoimmunity.

## Data Availability

The raw data supporting the conclusions of this article will be made available by the authors, without undue reservation.
